# Announcing a Nice Bump in the *Ochsner Journal* Impact Factor

**DOI:** 10.31486/toj.26.5064

**Published:** 2026

**Authors:** Ronald G. Amedee

**Affiliations:** Director of Clinical School and Professor, The University of Queensland Medical School, Ochsner Clinical School, New Orleans, LA; Editor-in-Chief, *Ochsner Journal*

*Celebrating good news reminds you that there's still hope—and the end of the story isn’t here yet.*

–*Branden Harvey*

The Fall 2026 issue of the *Ochsner Journal* contains one letter to the editor, two original research articles, one each of a quality improvement report and a review and contemporary update, and seven case reports and clinical observations.

The letter to the editor—“Focus on POCUS”—was penned by Koveleskie and Nossaman (Ochsner Anesthesiology) and complements a case report included in this edition: “Benefits of Point-of-Care Ultrasound for Assessing Gastric Contents.”

The first original research article is from Lutati, Brennan, Peterman, et al from Luminis Health in Annapolis, Maryland, and is entitled “Attention-Deficit/Hyperactivity Disorder in Anterior Cruciate Ligament Reconstruction Patients ≤25 Years of Age: A National Database Study of Prevalence and Outcomes.” This paper is followed by “Low-Cost 3D-Printed Temporal Bone Models for Surgical Simulation: A Pilot Study” authored by Tholen, Curtis, Sarkar, and Mankekar of the Ochsner BioDesign Lab and LSU Shreveport.

Our quality improvement article is entitled “Use of Safety Stand-Down Initiatives to Reduce Hospital Harm Events” and is by a patient safety–focused team led by Armin Schubert of Ochsner Anesthesiology.

The review and contemporary update submission in this issue is “Efficacy and Safety of the iStent infinite Trabecular Micro-Bypass Device for Glaucoma: A Systematic Review and Meta-Analysis” by Fischer, Duncombe, Okolie, and Caglianone (two students, an independent researcher from Nigeria, and a physician who works for the International Association of Providers of AIDS Care).

Case reports and clinical observations include “Avascular Necrosis Secondary to Traumatic Posterior Hip Dislocation” authored by Schoofs, Marshall, Willard, and Jones from Ochsner Orthopedics and Emergency Medicine. Next is a submission from Ochsner Anesthesiology, Ochsner Surgery, and Tulane Anesthesiology staff entitled “Transient Postoperative Myoclonus Following Hypothermic Circulatory Arrest.” “Atypical Visceral Involvement of Pyoderma Gangrenosum” was submitted by Radiology and Pathology staff at Hospital de la Santa Creu i Sant Pau in Barcelona, Spain. “Amputation for Complex Regional Pain Syndrome Type 1” came from staff at the Central Adelaide Local Health Network in Adelaide, Australia, and “Endometrial Carcinoma Unveiling Breast Carcinoma in a Postmenopausal Female: A Case of Synchronous Malignancy” was authored by Polavarapu, Pooja, Peddireddi, and Alahari from the Department of Pharmacology, Biochemistry and Experimental Therapeutics at LSU-New Orleans, and various institutions in India. “Isolated Tuberculous Testicular Abscess Without Systemic Involvement: A Rare Cause of Scrotal Swelling” was authored by three pathologists from Dr. Sampurnanand Medical College in Jodhpur, India.

In late June of this year, *Ochsner Journal* received information from Clarivate that the total number of *Journal* citations in calendar year 2025 was 1,870 (these citations are of articles published in 2023 and 2024), and the *Journal*’s impact factor had increased from 1.2 in 2024 to 1.5 in 2025. This impressive bump in the *Journal*’s impact factor is the good news that we are celebrating with you: Ochsner Health staff, our authors and reviewers, and our readers throughout the world. We are immensely proud of this accomplishment which is in large part due to the efforts of one individual, Kathleen McFadden, who serves as the managing editor of this quarterly publication. I also wish to take this opportunity to thank our reviewers for their diligent efforts and the many authors who submit their work for consideration of publication following the peer-review process. The *Journal* is always in need of additional reviewers and top-quality article submissions. Please visit our website at ochsnerjournal.org if you are inclined to assist us in continually improving and enhancing this publication or call Kathleen at (504) 842-7398.

